# High-temperature analysis of optical coupling using AlGaAs/GaAs LEDs for high-density integrated power modules

**DOI:** 10.1038/s41598-022-06858-5

**Published:** 2022-02-24

**Authors:** Syam Madhusoodhanan, Abbas Sabbar, Huong Tran, Pengyu Lai, David Gonzalez, Alan Mantooth, Shui-Qing Yu, Zhong Chen

**Affiliations:** Department of Electrical Engineering, University of Arkansas, Fayetteville, AR 72701 USA

**Keywords:** Electrical and electronic engineering, Characterization and analytical techniques

## Abstract

A low-temperature co-fired ceramic (LTCC)-based optocoupler design is demonstrated as a possible solution for optical isolation in high-density integrated power modules. The design and fabrication of LTCC based package are discussed. Commercially available aluminum gallium arsenide/gallium arsenide (AlGaAs/GaAs) double heterostructure is used both as emitter and photodetector in the proposed optocoupler. A detailed study on the electroluminescence and spectral response of the AlGaAs/GaAs structure is conducted at elevated temperatures. The material figure of merit parameter, D*, is calculated in the temperature range 77–800 K. The fabricated optocoupler is tested at elevated temperatures, and the results are presented.

## Introduction

Third-generation semiconductor materials such as silicon carbide (SiC) and gallium nitride (GaN) improved the operations and the performances in terms of power capability, switching frequencies and temperature tolerance in the field of power electronics^1–3^. While GaN devices are mainly preferred for applications below 500 V, SiC is the ideal material of choice for power modules with higher voltage and current ratings. Commercially available SiC devices, rated 900 V and above, with a chip size spanning few millimeters, enables the development of high-density power modules with a low form factor^4^. A low form factor power electronic system design is vital in size and/or weight-sensitive applications such as aeronautics, hybrid motor drives, and space exploration^5–7^. The miniaturization of the wide bandgap-based power electronic system is mainly made possible by utilizing the higher junction temperature operations and SiC devices, thus eliminating a bulky cooling system^8,9^. Integrating the gate driver and protection circuity to the power module further reduces the system’s form factor and improves the operational frequencies^10–13^. In this integrated power module, the gate driver’s low-voltage passive components and the protection circuitry are placed close to the power devices, making them exposed to similar environmental conditions as the power devices. Therefore, the integrated power modules’ volumetric reduction is now limited by passive components’ temperature capabilities in the gate driver circuitry.

To fully utilize the volumetric improvements ensured by using SiC technology in the power electronics system, the focus should be on improving the packaging’s thermal capability and the passive components in the design. Most of the passive components, such as resistors, capacitors, and magnetics, are rated up to a maximum temperature of 300 °C^14,15^. However, the high temperature rated magnetics, such as isolation transformers, are bulky in design, leading to comparatively lower power density modules. Therefore, the design of a low form factor integrated power modules with bulky isolation transformers is challenging. A low form factor optical isolation system often replaces bulky transformers in power modules. However, in the integrated power module design, the optical isolation system should be reliable at elevated temperatures (> 200 °C). A detailed study on the temperature performance of the optocouplers currently available in market is published elsewhere^16^. With the development of a high-temperature optical isolation system, i.e., optocouplers, volumetric issues associated with the magnetic isolation system can be avoided while simplifying the gate circuitry.

In this paper, we report the high-temperature (> 250 °C) operation of double heterojunction (DH) AlGaAs/GaAs light-emitting diodes (LEDs) as emitter as well as a photodetector. We also propose a primitive design and fabrication of the high-temperature optocoupler using LTCC technology. The rationale for selecting the AlGaAs/GaAs structure can be explained by (i) overlapping emission and absorption spectra of AlGaAs/GaAs LEDs at room temperature (ii) superior temperature stability and spectral response GaAs devices over traditional Si devices^17^. The LTCC package was selected due to its higher temperature stability and the ease of integrating into power module designs^18,19^. Temperature-dependent electroluminescence (EL) and spectral response measurements were carried out on bare die devices to eliminate the failures from packaging materials. The internal quantum efficiency (IQE) and specific detectivity of the device are studied for different temperatures. The output current as a function of the input current of the LTCC-based optocoupler is measured and analyzed at elevated temperatures. This study demonstrates the development of high-temperature optocouplers and examines the possibility of driving miniaturization trends in high-density power modules by eliminating the volumetric issues in design architecture.

## Methods

AlGaAs/GaAs DH infrared LEDs with overlapping emission and absorption spectrum at room temperature are selected for high-temperature optical and electrical studies. Bare-die devices, with a dimension of 960 × 960 µm^2^, are used to avoid degradation of packaging media and forming lenses at high temperatures. Commercial LED/Photodetector is sourced from Marktech Optoelectronics, Model No: OPC7000-21. High-temperature characterization of the LEDs was carried out in a Janis ST-100 cryostat. Temperature- and intensity-dependent electroluminescence (T-IDEL) measurements were carried out using a Horiba 550 spectrometer integrated with a photomultiplier tube (PMT)^20^. The spectral responses of the structure were obtained using a tunable monochromator with a 250 W, 24 V tungsten halogen lamp. The incident power on the LEDs is measured using a calibrated silicon detector. A lock-in amplifier is used to measure the photocurrents. The dark current–voltage (I–V) characteristics in the temperature range 77 K–800 K is measured using a Keithley 236 source measurement unit (SMU).

**Figure 1:**
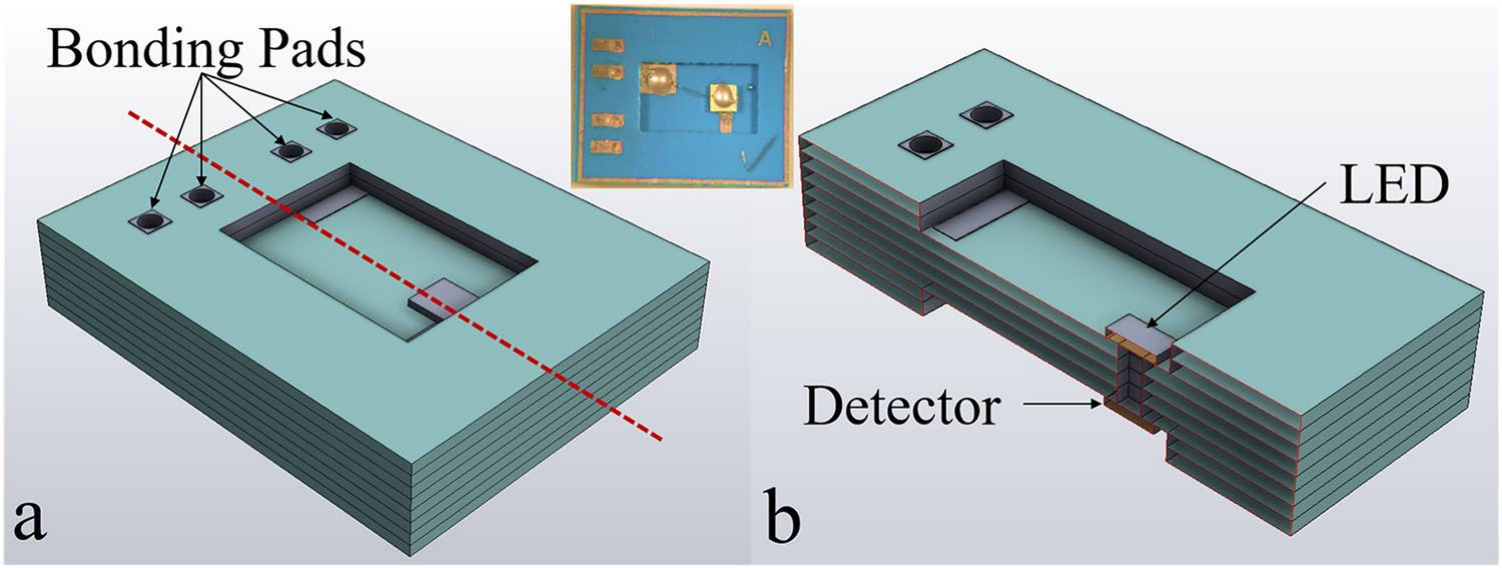
(**a**) 3D CAD design of the LTCC based optocoupler, (**b**) cros sectional view. Inset shows the fabricated device.

The bare die devices were integrated into an LTCC-based optocoupler package for high-temperature measurements. The high-temperature measurements were performed in the Janis ST-100 cryostat. The LTCC package is fabricated at the University of Arkansas (UA) High-Density Electronic Center (HiDEC) facility. Figure 1 shows the 3D design of the LTCC package; the inset shows the fabricated device. The package comprises eight layers of DuPont GreenTape 951 with a thickness of 254 um. Gold traces with a width of 0.60 mm are screen printed along with 0.50 × 0.50 mm^2^ vias are created on the Dupont GreenTape for the electrical connections. Ferro 4007 Brazeable Au Conductor paste is used for trace printing. The separation between LED and detector is around 1 mm. After firing, the total volume of the package is around 10*8*1.7 mm^3^.

## Results and discussion

**Figure 2:**
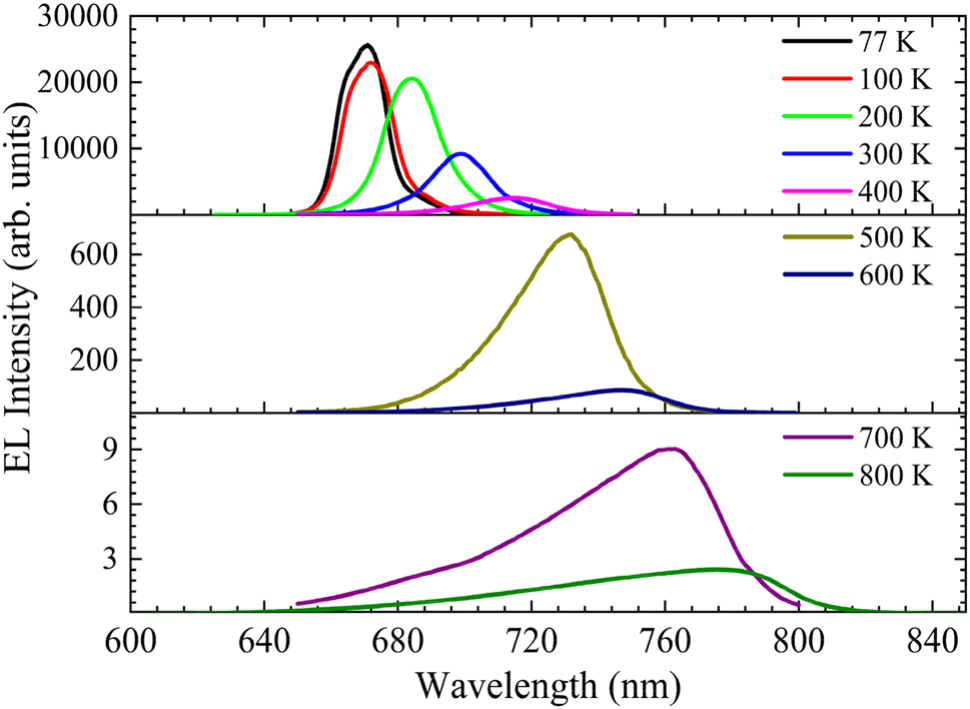
The EL spectra of the AlGaAs/GaAs DH is measured at a current density of 0.325A/cm^2^ for different temepratures.

The evolution of the EL spectra of the AlGaAs/GaAs DH structure over the temperature is shown in Fig. 2 with an injected current density of 0.325 A/cm^2^. A decrease in the EL intensity and a spectrum broadening are observed at higher temperatures. The DH structure’s spectral peak exhibits redshift at elevated temperatures from 670 nm at 77 K to 784 nm at 800 K. The shift of the spectral peak is attributed to the bandgap narrowing effect at elevated temperatures^21^. Significant reduction in the EL intensity is observed when the temperature is increased from 77 to 800 K. A four orders of magnitude reduction in the EL intensity at elevated temperatures indicates a severe drop in IQE. However, the temperature droop, i.e., the reduction in IQE due to high temperatures, is also dependent on the injected current density. A detailed understanding of the IQE behavior w.r.t the injected current density enables the optimal selection of biasing conditions for LEDs to achieve a minimal drop in IQE at elevated temperatures.

**Figure 3:**
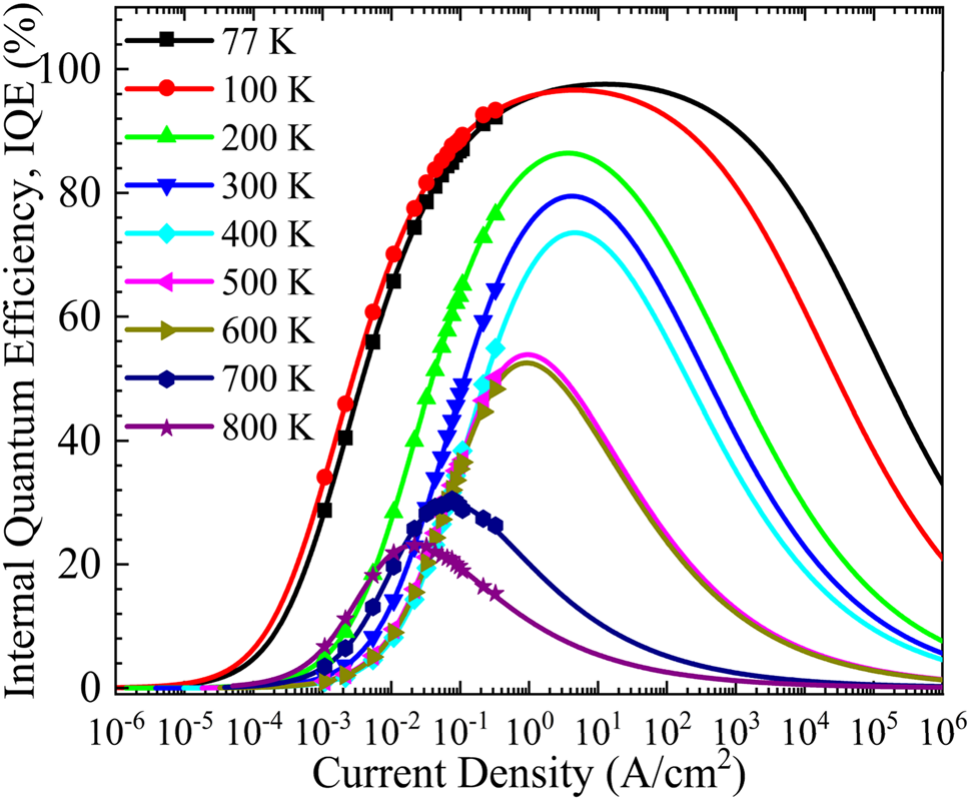
Internal quantum efficiency is plotted as a function of current density at different temperatures.

**Figure 4:**
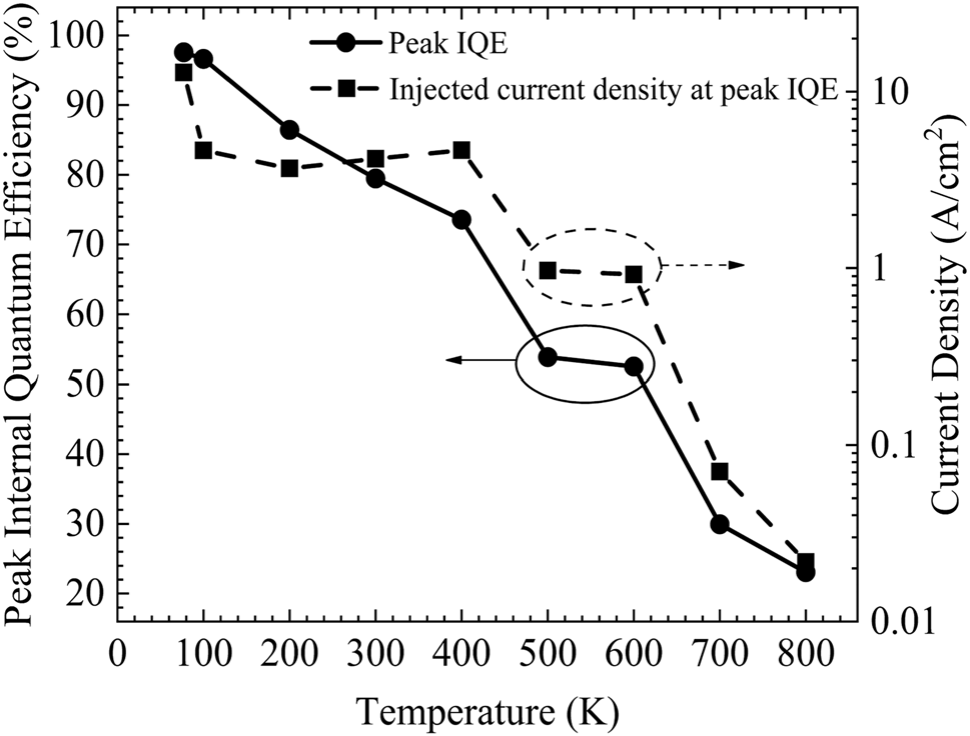
The peak IQE and the corresponding injected current density are extracted and plotted as a function of temperatures.

ABC model is used to extract the of AlGaAs/GaAs LEDs at higher temperatures^22^. Figure 3 shows IQE as a function of injected current density for different temperatures. The dots are the experimental points, and the solid lines are extracted using the ABC model. The efficiency drops at higher injected current densities, often called current droop, leads to the dome-like structure of the IQE curves. The current droop behavior is due to the enhanced Auger recombination mechanism, a non-radiative recombination mechanism, which suppresses the radiative recombination at higher injected current densities^23^. It is observed that at higher temperatures, auger recombination starts to dominates over radiational recombination at relatively lower injected current densities leading to the peak IQE occurs at lower injected current densities. The drop in the injected current densities to achieve peak IQE at higher temperatures can also be attributed to the increase in charge injection efficiencies and transport resistance due to heat accumulation. The LED shows a peak quantum efficiency of 97.58% at 77 K. The peak IQE of the device decreases with an increase in temperature. At 800 K, the device exhibits a peak IQE (P_IQE_) of 23.08%. The extracted values of the P_IQE_ and corresponding injected current densities (jP_IQE_) are plotted as a function of temperature, as shown in Fig. 4. The device exhibits a linear drop in IQE from 77 to 400 K, with approximately a reduction of 7% IQE in every 100 K. Increasing the temperature beyond 400 K shows a severe reduction in IQE from 73.57% at 400 K to 53.83% at 500 K. The peak current respective to the peak IQE follows a similar trend that of P_IQE_ with an increase in temperature. The jP_IQE_ reduced from 12.85 A/cm^2^ at 77 K to 21.77 mA/cm^2^ at 800 K. Low jP_IQE_ enables the device to work at peak IQE with minimal self-heating.

**Figure 5:**
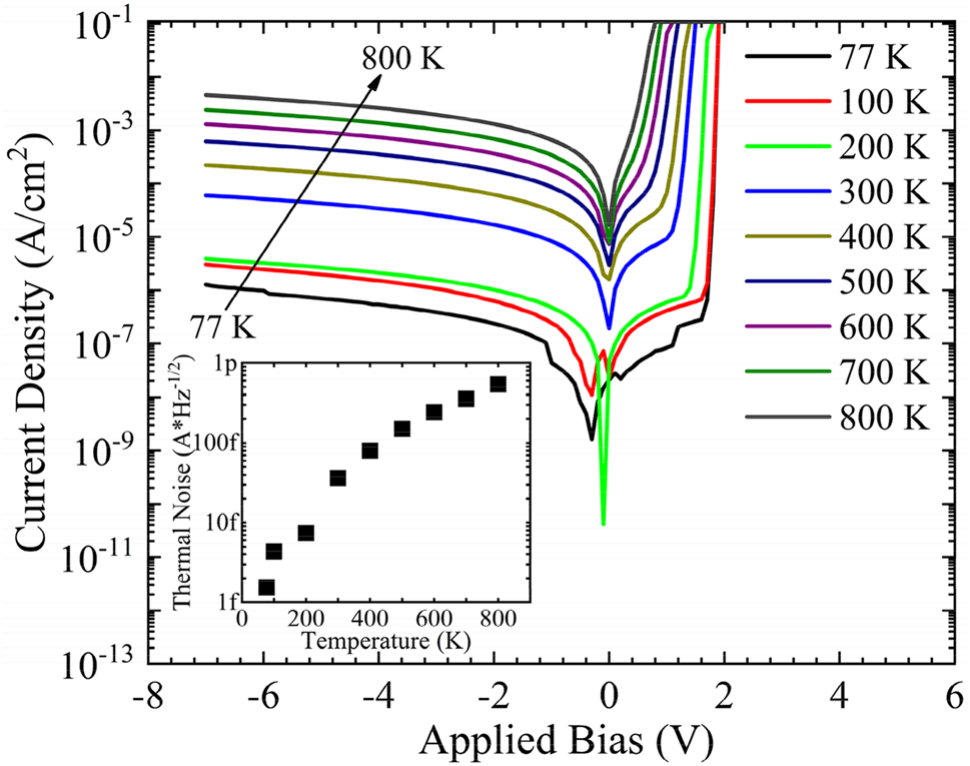
I–V characteristics of the AlGaAs/GaAs DH for temperatures from 77 to 800 K. Inset shows the evolution of thermal noise.

**Figure 6:**
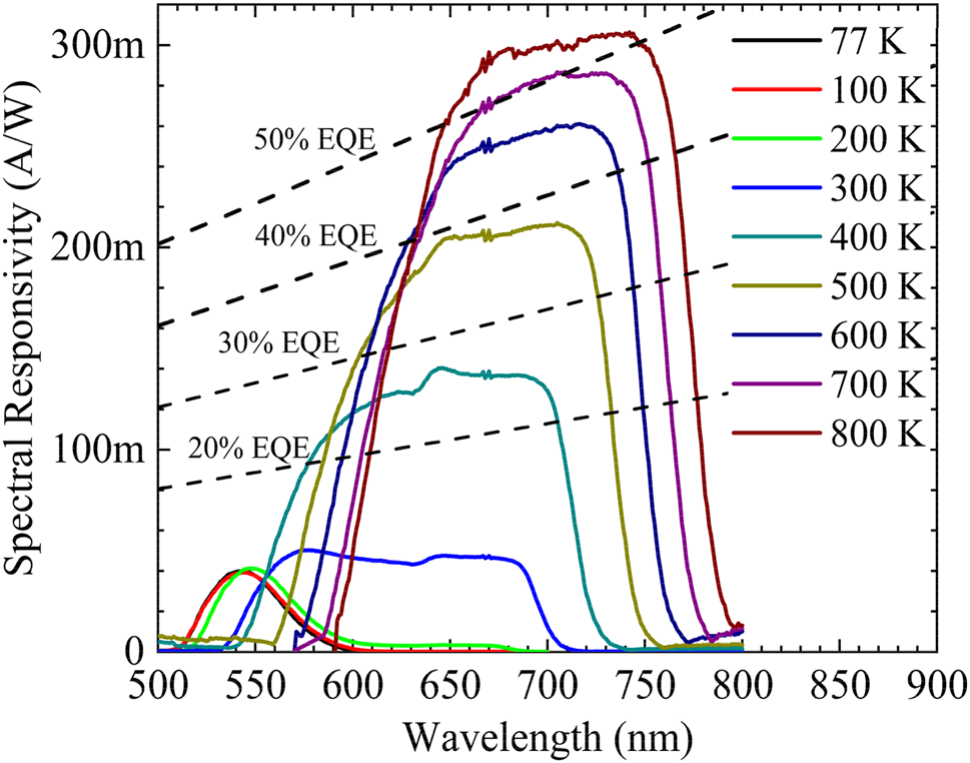
Spectral responsivity of AlGaAs/GaAs DH for different temperatures at zero bias. The respective EQE is showed as dashed lines.

Stable operation at 800 K and overlapping emission and absorption spectra lead to a detailed study on the photodetection characteristics of the AlGaAs/GaAs DH structure. Figure 5 shows the dark IV characteristics of the structure from 77 to 800 K; the inset shows the evolution of thermal noise with temperature. An exponential rise in thermal noise is observed from the structure at elevated temperatures. Three orders of magnitude change in the leakage current is observed when the temperature is increased from 77 to 800 K. A sudden increase in the leakage current as well as the thermal noise at 300 K is attributed to the thermal ionization of carriers from deep traps and trap assisted tunneling process^24^. The spectral response of the device at zero biased condition is shown in Fig. 6 for different temperatures; the dashed line represents spectral responsivity with a specified external quantum efficiency (EQE) across the measured wavelength. An enhanced spectral response is observed at elevated temperatures. The spectral response curves exhibit a large redshift in the spectral response peak and the detection edge at elevated temperatures. An increase in temperature shifts the absorption spectrum of the structure to longer wavelengths by reducing the effective bandgap.

**Figure 7:**
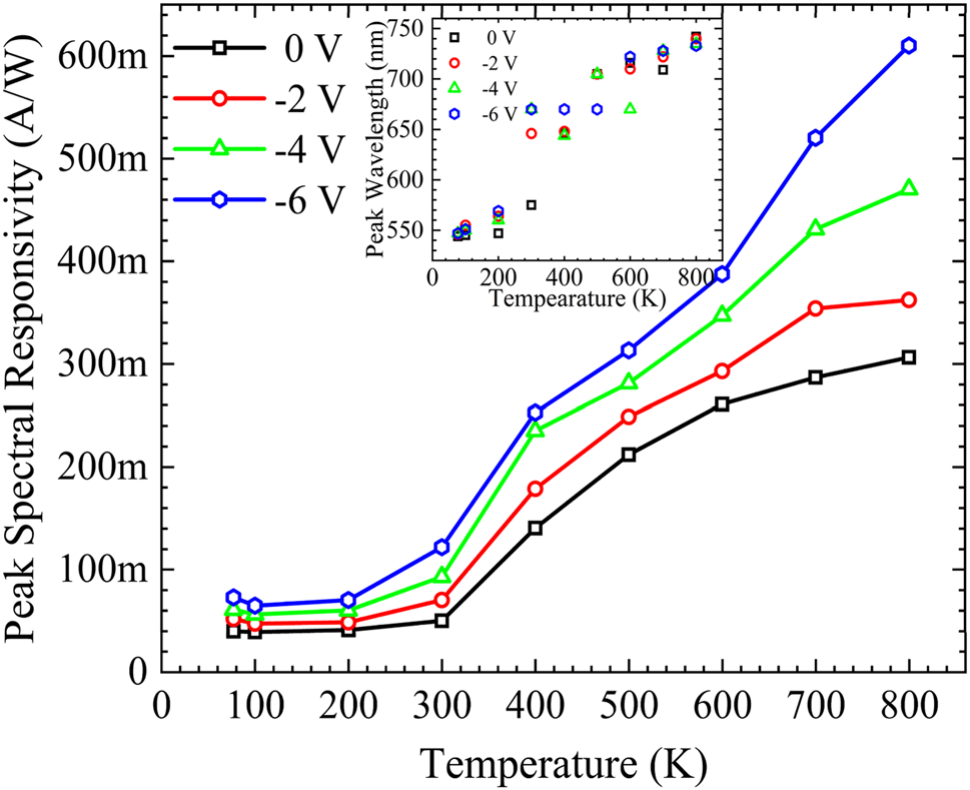
Peak spectral responsivity of the AlGaAs/GaAs DH for different biased conditions. Inset shows the redshift of peak responsivity with respect to temperature.

Figure 7 represnts the temperature dependant behaviour of the peak spectral responsivity of the structure at different biased conditions; inset shows the respective wavelength at which the spectral peak is observed. The structure shows a relatively small increment in the spectral peak at lower temperatures. Increasing the temperature above 300 K results in an exponential change in the responsivity. The rapid increase in the spectral responsivity above 300 K is attributed to (i) shift in the absorption spectrum towards higher wavelengths due to bandgap shrinkage, as shown in the inset of Fig. 7 (ii) reduced absorption rate at higher wavelength leads to more photons reaching the active area resulting in a higher responsivity. Biasing the structure with higher voltages leads to a broader space charge region resulting in enhanced spectral responsivity, as shown in Fig. 7. At elevated temperatures, the spectral peak responsivity tends to saturate at 800 K for lower bias curves. However, no sign of saturation in the spectral peak response is visible for higher biased curves, even at 800 K.

**Figure 8:**
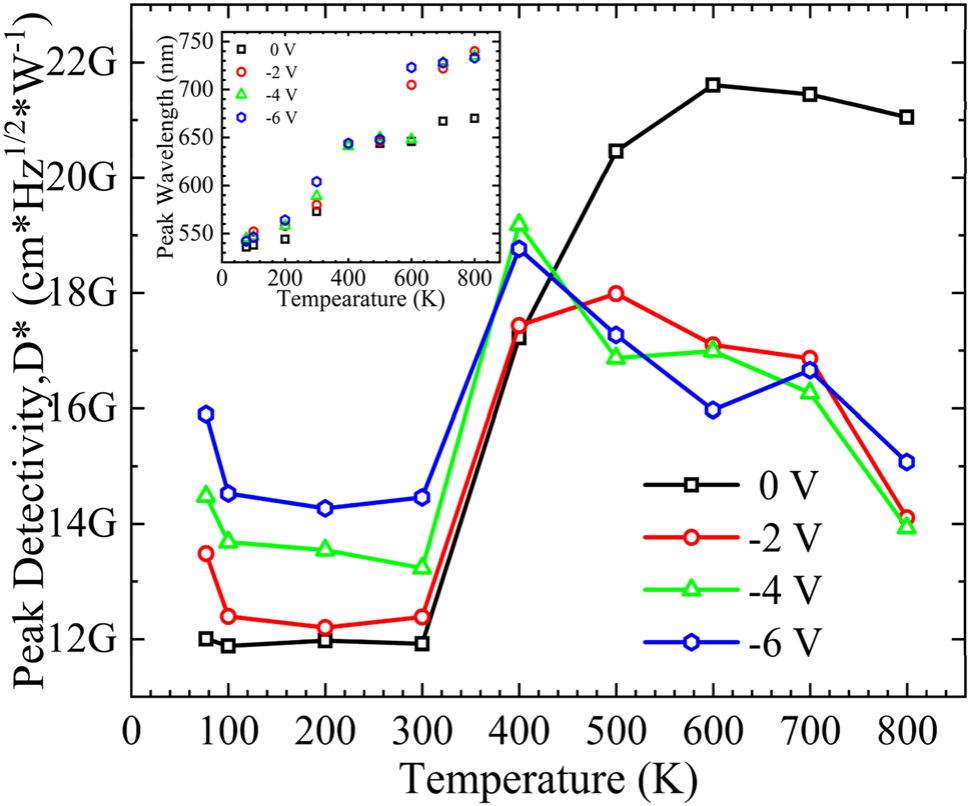
The peak specific detectivity at different biased conditions. Inset shows the redshift corresponding to the peak specific detectivity.

The specific detectivity, D*, of the structure is extracted for different temperatures and biased conditions to quantify the performance of the photosensitivity of the structure. Figure 8 shows the peak-specific detectivity as a function of temperature for different biased conditions; the inset shows the wavelength at which peak detectivity is observed. The structure exhibits slight variation in the D* at lower temperatures. At zero bias, the D* of the structure increased from 12G Jones (cmHz^1/2^ W^-1^) at 300 K to 17G Jones at 400 K. A rapid increase in the D* at 400 K is attributed to the increase in the spectral responsivity due to a significant redshift with minimal increase in the leakage current and thermal noise. The structure showed a peak D* of 22G Jones at 600 K with zero bias. The D* starts to decline at temperatures above 600 K. However, at biased conditions, the D* starts to decline at temperatures above 400 K. Although the structure shows a linear increase in spectral responsivity with bias voltage at elevated temperatures, higher noise current at biased conditions leads to a reduced D*. At elevated temperatures, the photosensitive performance of the structure is limited by bias-induced internal noise. A superior noise performance at zero bias at elevated temperatures suggests a higher chabge in noise levels compared to responsivity when the applied bias increases.

**Figure 9:**
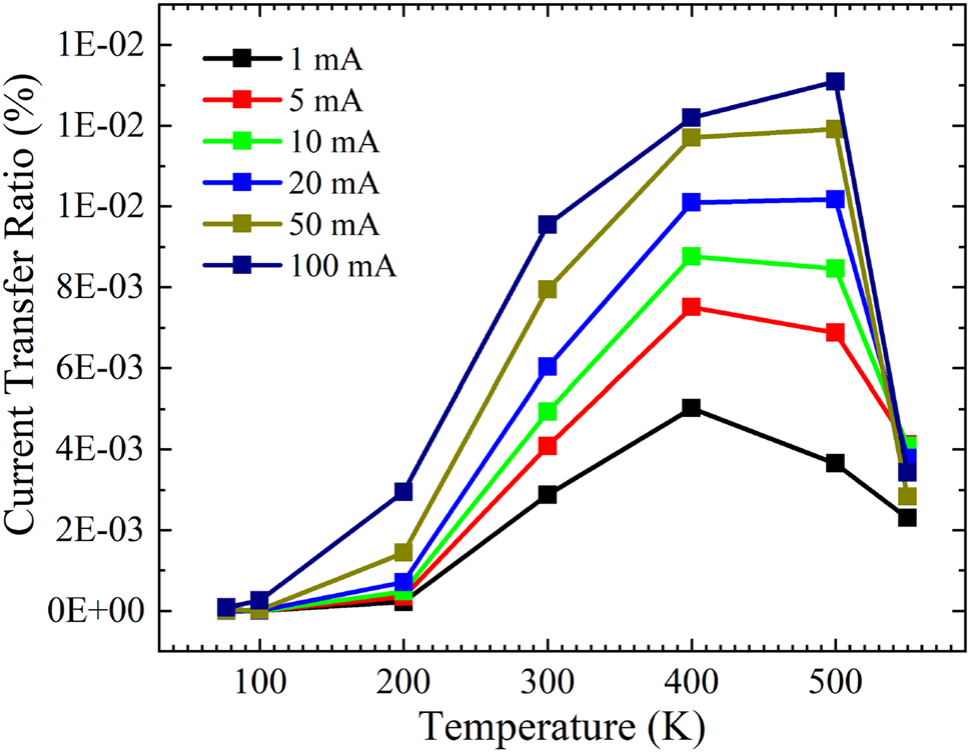
The output current of the fabricated LTCC based optocoupler is plotted as a function of the input current for different temperatures.

High-temperature analysis of the emitter and photodetector operation of the discrete devices is followed by the design and fabrication of the LTCC package. The individually tested devices are integrated into the LTCC package, creating an optocoupler and testing the optical coupling efficiency at elevated temperatures. The current transfer ratio (CTR) of the integrated LTCC based optocoupler for different input current is shown in Fig. 9 without any external amplification. The input current to the LED is varied from 1 to 100 mA, and the detector is biased at zero voltage. The device shows comparatively low CTR values below 200 K. A lower CTR values at temperatures at 200 K and below are attributed to the reduced spectral response of the AlGaAs/GaAs DH structure. Although the LED performance degrades with an increase in temperature, the spectral response of the structures improves at elevated temperature leading to higher CTR values at temperatures above 200 K. The CTR value of the structure starts to degrade at temperatures above 500 K. A reduced CTR value is recorded at 550 K. Temperatures above 550 K, the structure failed to output any photocurrent. It is concluded that a higher degradation of the EL intensity at temperatures above 500 K causes the CTR to drop. It is observed that the rate of degradation of the EL intensity is dominating over the enhanced spectral responsivity at higher temperatures. Stable CTR values in the temperature range of 300 to 500 K are promising in developing high-temperature optocouplers for future high-density integrated power modules.

## Conclusion

A high-temperature optocoupler based on LTCC packaging is demonstrated as a possible solution for optical isolation in future high-density power modules. An LTCC based optocoupler package is fabricated using eight layers of DuPont GreenTape 951 with a thickness of 254 um. After firing, the total volume of the package is around 10*8*1.7 mm^3^. AlGaAs/GaAs DH devices are integrated into the LTCC package, both as emitter and photodetector, with a separation of 1 mm. Detailed analysis of the high-temperature behavior of AlGaAs/GaAs DH structure as both emitter and photodetector is conducted over the temperature range from 77 to 800 K. While the EL spectra of the discrete AlGaAs/GaAs structure reduces with temperature, and enhanced spectral response is observed at elevated temperatures. The LED structure shows a significant red shift in the EL spectra, as well as the detection range from 670 nm at 77 K to 784 nm at 800 K. The LED structure exhibits an IQE of 22% at 800 K. The photosensitivity of the structure is quantified using the material figure of merit parameter, D*. A peak detectivity of 22G Jones at zero bias is observed at 600 K. The fabricated optocoupler shows a stable operation in the temperature range of 300–550 K.
